# Alpha-Fetoprotein, Identified as a Novel Marker for the Antioxidant Effect of Placental Extract, Exhibits Synergistic Antioxidant Activity in the Presence of Estradiol

**DOI:** 10.1371/journal.pone.0099421

**Published:** 2014-06-12

**Authors:** Hye Yeon Choi, Seung Woo Kim, BongWoo Kim, Hae Na Lee, Su-Jeong Kim, Minjung Song, Sol Kim, Jungho Kim, Young Bong Kim, Jin-Hoi Kim, Ssang-Goo Cho

**Affiliations:** 1 Department of Animal Biotechnology, Animal Resources Research Center, and Incurable Disease Animal Model and Stem Cell Institute (IDASI), Konkuk University, Seoul, Republic of Korea; 2 Department of Life Science, Sogang University, Seoul, Republic of Korea; Institut Jacques Monod, France

## Abstract

Placenta, as a reservoir of nutrients, has been widely used in medical and cosmetic materials. Here, we focused on the antioxidant properties of placental extract and attempted to isolate and identify the main antioxidant factors. Porcine placental extracts were prepared through homogenization or acid hydrolysis, and their antioxidant activity was investigated in the human keratinocyte HaCaT cell line. Treatment with homogenized placental extract (H-PE) increased the cell viability of H_2_O_2_-treated HaCaT cells more than two-fold. H-PE treatment suppressed H_2_O_2_-induced apoptotic and necrotic cell death and decreased intracellular ROS levels in H_2_O_2_-treated HaCaT cells. The antioxidant factors in H-PE were found to be thermo-unstable and were thus expected to include proteins. The candidate antioxidant proteins were fractionated with cation-exchange, anion-exchange, and size-exclusion chromatography, and the antioxidant properties of the chromatographic fractions were investigated. We obtained specific antioxidant fractions that suppressed ROS generation and ROS-induced DNA strand breaks. From silver staining and MALDI-TOF analyses, alpha-fetoprotein (AFP) precursor was identified as a main marker for the antioxidant effect of H-PE. Purified AFP or ectopically expressed AFP exhibited synergistic antioxidant activity in the presence of estradiol. Taken together, our data suggest that AFP, a serum glycoprotein produced at high levels during fetal development, is a novel marker protein for the antioxidant effect of the placenta that exhibits synergistic antioxidant activity in the presence of estradiol.

## Introduction

Skin is constantly exposed to various chemical, physical, and dietary pollutants [1]. When the skin is constantly exposed to pollutants, reactive oxygen species (ROS) are generated that cause a variety of skin disorders [2], [3]. At low levels, ROS take part in the regulation of numerous cellular processes, including cell proliferation, apoptosis, immune responses, and cell differentiation. Overproduction or inadequate removal of ROS can result in oxidative stress, leading to altered metabolism, dysregulated signal transduction events, and biomolecular damage to lipids, proteins, and DNA, all of which contribute to pathological changes in cell and tissue function [4]. Thus, ROS play important roles in aging [5] and in the pathogenesis of many diseases [5], [6]. ROS are generally very small molecules and are highly reactive because of the presence of unpaired valence shell electrons. At the molecular level, ROS alter cell signaling and trigger apoptotic processes [7]. There are several types of ROS, such as superoxide (O_2_ ˙), hydrogen peroxide (H_2_O_2_), and the highly reactive hydroxyl radical (OH ˙), which are formed by transferring physical or chemical energy to molecular oxygen (O_2_) [7], [8]. Among the various ROS, H_2_O_2_ plays an important role because it is generated from nearly all sources of oxygen free radicals. H_2_O_2_ is reported to generate hydroxyl radicals that induce lipid peroxidation within exposed cells, leading to DNA damage and cell death [9], [10]. Moreover, these oxygen free radicals have been implicated in the onset of skin diseases [11], [12]. The identification of non-toxic antioxidants that prevent oxidative stress reactions and protect cells against the adverse effects of pro-oxidants has been the focus of extensive research [5], [13]. Many antioxidant substances have been characterized, including vitamin C, vitamin E, superoxide dismutases, nicotinamide adenine dinucleotide phosphate (NADPH), and polyphenols [14], [15].

Placental extracts (PEs) have been used for years as a folk remedy, for example, in wound healing, and as a cosmetic in many countries [16], [17]. Many studies have also described the wound healing and anti-inflammatory properties of PE [3], [5]–[7], [13], . PE has numerous bioactive components, such as anti-oxidants, nutrients, oxygen, and hormones [1], [3], [27]. One of the most important roles of the placenta is to protect the embryo(s) from oxidative stress [39], [48]. Therefore, PE has antioxidative properties. The major antioxidant components in PE are uracil, tyrosine, phenylalanine, and tryptophan. Approximately 59% of the antioxidant effect of PE can be attributed to these components [49].

In this study, we focused on the antioxidant property of PE and searched for novel antioxidant factors. We prepared PEs in two ways, by acid hydrolysis or homogenization. The antioxidant characteristics of acid-hydrolyzed placental extracts (A-PEs) and homogenized placental extracts (H-PEs) were investigated in the human keratinocyte HaCaT cell line.

## Materials and Methods

### Cell culture and DNA transfection

The human keratinocyte HaCaT cell line (a gift from Professor Ick-Hwan Kim, Korea University, Korea) was cultured in Dulbecco's modified Eagle's medium (DMEM; Invitrogen, Carlsbad, CA, USA) supplemented with 10% fetal bovine serum (FBS; HyClone, Logan, UT, USA) and 100 U/ml penicillin/streptomycin (HyClone) and incubated at 37°C with 5% CO_2_. HaCaT cells were treated with hydrogen peroxide (H_2_O_2_; Sigma-Aldrich, Saint Louis, MO, USA) diluted in serum-free media.

Cells were transfected using Lipofectamine 2000 (Invitrogen) according to the manufacturer's instructions. Briefly, for DNA transfection, HaCaT cells were incubated overnight at a density of 2×10^5^ cells per 60-mm culture dish and transfected with expression vectors using Lipofectamine 2000. Stable cell lines were selected by growth in the presence of 800 µg/ml G418 (Sigma-Aldrich). HaCaT cells expressing GFP-tagged alpha-fetoprotein (AFP) were used to confirm the antioxidant potency.

### Preparation of placental extracts from porcine placenta

Porcine placental extracts were prepared from multiple porcine placentas, which were provided by ChoongAng Biotech (Kyungkido, Korea) [19]. To prepare H-PE, multiple porcine placentas were obtained from uncomplicated pregnancies immediately after delivery. The umbilical cord and amnion were discarded, and the remaining tissues were washed completely in ice-cold 0.9% NaCl (Amresco, Solon, OH, USA) to remove all traces of blood. Homogenates were prepared using phosphate-buffered saline (PBS; HyClone) with a knife homogenizer (Daesung, Kyungkido, Korea) and Polytron Homogenizer (ART Prozess- & Labortechnik GmbH & Co. KG, Müllheim, Germany). A-PE was prepared according to the procedure provided by ChoongAng Biotech [19]. Briefly, several porcine placentas were hydrolyzed with 6 N HCl (Dae Jung, Kyungkido, Korea). Next, the protein hydrolysis enzymes were inactivated at 110°C for 5 hr, and the hydrolysates were filtered through activated carbon. The lipids were then removed by mixing with calcium and phosphate salts and by filtering through two types of absorbents. The hydrolysates were neutralized with 10 N NaOH (Sigma-Aldrich) in an ion-exchange resin and filtered. Finally, the porcine placental extract was sterilized at 121°C for 15 min.

### Preparation of heat-treated placental extracts

H-PEs in one group were heated at temperatures ranging from 55°C to 60°C for 1 hr, and H-PEs in the other group were heated at 60°C for 1 to 60 min. After heating, the extracts were immediately placed on ice and then centrifuged at 5000 rpm for 15 min.

### Cell viability assay

Cell proliferation and viability were assessed with MTT assays (Cell Counting Kit 8; CCK-8; Dojindo Molecular Technologies, Rockville, MD, USA). Cells were plated at a density of 1×10^4^ cells/well in 96-well plates and treated with MTT solution [CCK-8 solution: 2-(2-methoxy-4-nitrophenyl)-3-(4-nitrophenyl)-5-(2,4-disulfophenyl)-2H-tetrazolium, monosodium salt] for 4 hr at 37°C. Cell viability was measured with an enzyme-linked immunosorbent assay (ELISA) reader (Bio-Rad, Hercules, CA, USA) at 450 nm.

### Detection of apoptotic or necrotic cells

Cells were stained with SYTOX and Hoechst 33342 (Life Technologies, Carlsbad, CA, USA) to identify the type of cell death [16]. Cells were incubated with a mixture of 5 µM SYTOX and 5 µg/ml Hoechst 33342 for 5 min at 37°C and observed using an inverted fluorescent microscope (Carl Zeiss, Oberkochen, Germany). Nuclear condensation and fragmentation was evident in the Hoechst 33342-stained apoptotic cells, and the percentage of apoptotic cells was calculated as the ratio of apoptotic cells to total counted cells. The SYTOX-stained necrotic cells were detected by FACS analysis (FACScan; Becton Dickinson, La Jolla, CA, USA). Briefly, cells were washed with serum-free media and trypsinized. Cells were then harvested by centrifugation for 15 min at 1000 rpm. The collected cells were washed with PBS and analyzed by FACS.

### Detection of intracellular ROS generation

For measurement of intracellular ROS levels, cells were incubated with 10 µM 2′-7′-dichlorofluorescin diacetate (H_2_DCFH-DA; Invitrogen) for 30 min at 37°C in the dark. Fluorescence-stained cells were analyzed by FACS analysis using Cell Quest software (Becton Dickinson) for acquisition and analysis. The antioxidant capacity of the placental extracts was estimated from the intracellular ROS accumulation after H_2_O_2_ challenge. N-acetylcysteine (NAC; Sigma-Aldrich) and estradiol (Sigma-Aldrich), well-known antioxidant reagents [20]–[22], were used as positive controls.

### High-performance liquid chromatography (HPLC)

For high-performance strong ion-exchange chromatography, a pre-packed HiTrap SPFF column and Superdex 200 10/300 GL column were purchased from GE Healthcare Life Sciences (Princeton, NJ, USA). An ACME 9000 HPLC System (Young Lin, Kyungkido, Korea) equipped with a binary solvent delivery system and a UV/Vis detector was used. The solvents were delivered by an ACME 9000 gradient pump. The gradient conditions were programmed using an Autochro-3000 HPLC Data System. For cation-exchange HPLC, homogenates were loaded directly on a HiTrap SP FF strong cation-exchange column (GE Healthcare Life Sciences) equilibrated with 0.05 M sodium phosphate (pH 8.0, Buffer A; Sigma-Aldrich). The homogenates were eluted at a flow rate of 1 ml/min with a step gradient program from buffer A to buffer B [0.05 M sodium phosphate, 1 M NaCl (Sigma-Aldrich), pH 8.0]. For anion-exchange chromatography, homogenates were loaded directly on a HiTrap Q FF strong cation-exchange column (GE Healthcare Life Sciences) equilibrated with 0.02 M Tris-HCl (pH 7.4; Buffer C; Sigma-Aldrich). The homogenates were eluted at a flow rate of 1 ml/min with a step gradient program from buffer C to buffer D (0.02 M Tris-HCl, 1 M NaCl, pH 7.4). For size-exclusion chromatography, homogenates were loaded onto a Superdex 200 10/300 GL size-exclusion chromatography column (GE Healthcare Life Sciences), which had been pre-equilibrated with 0.05 M sodium phosphate (pH 7.0) and 0.15 M NaCl. The column was developed with the same buffer at a flow rate of 0.5 ml/min. The molecular mass of the proteins was estimated by comparison with the elution rate of standard molecules (Bio-Rad). The protein elution profile was monitored with a UV/Vis detector at 280 nm. The protein concentrations were presented as the voltage (mV).

### Western blotting and silver staining

Cells were washed 3 times in ice-cold PBS, scraped from the dishes, and collected in extraction buffer [1% Triton X-100 (Amresco), 100 mM Tris-HCl, p 7.5, 10 mM NaCl, 10% glycerol (Amresco), 50 mM sodium fluoride (Sigma-Aldrich), and 1 mM phenylmethylsulfonyl fluoride (PMSF; Sigma-Aldrich)]. After incubation on ice for 20 min, lysates were centrifuged, and proteins in the supernatants were quantified using the Bradford Protein Assay Reagent (Bio-Rad). Equal amounts of protein were separated on an 8%–12% SDS-PAGE gel and transferred electrophoretically onto a nitrocellulose membrane (0.2 mm; Protran, Germany). Membranes were blocked in 5% nonfat dry milk (Sigma-Aldrich) and 0.1% Tween-20 (Amresco) in Tris-buffered saline and probed with primary antibody. Antibodies against anti-poly (ADP-ribose) polymerase (PARP), human AFP, and anti-beta-actin were obtained from Santa Cruz Biotechnology (Dallas, TX, USA). Antibody-antigen complexes were incubated with goat anti-mouse IgG- or goat anti-rabbit IgG-peroxidase conjugates and detected using an enhanced chemiluminescence (ECL) kit (GE Healthcare Life Sciences).

Gels were silver stained using a silver staining kit (GE Healthcare Life Sciences) according to the manufacturer's recommendations. Briefly, after SDS-PAGE, the gel was fixed with 30% ethanol and 10% acetic acid for 1 hr and then incubated in sensitizing solution with 30% ethanol, 0.2% sodium thiosulfate, and 6.8% sodium acetate for 1 hr. After washing 4 times with distilled water for 15 min each, the gel was stained with 0.25% silver nitrate solution for 1 hr and washed again 2 times for 1 min each. The gel was developed in 2.5% sodium carbonate and 0.04% formaldehyde until the protein bands were visible. To stop development, 1.46% EDTA solution was added.

### Reverse transcriptase-PCR analysis

Total RNA was extracted from the cells using TRIzol reagent (Gibco-BRL, Bethesda, MD, USA) according to the manufacturer's protocol. The extracted RNA was subsequently treated with MMLV reverse transcriptase (Promega, Madison, WI, USA). The first-strand cDNA was prepared from 2.5 µg of total RNA using reverse transcriptase and an oligo-dT primer. cDNA was amplified by PCR with DNA primers and Taq polymerase (Promega). PCR was performed using specific primers for human AFP (forward, 5′- CTGCAATTGAGAAACCCACTG-3′ and reverse, 5′- TTCCCTCTT CACTTTGGCTG-3′) and GAPDH (forward, 5′-GGGAAGAGTCAACGGATTTGGTCGT-3′ and reverse, 5′-GGGAATTGATTTTGGAGGGATCTCG-3′). The cycling conditions were 94°C for 5 min, followed by 35 cycles of 94°C for 30 s, 55°C for 30 s, and 72°C for 1 min, and a final extension at 72°C for 7 min. All samples were run in triplicate. The PCR products were analyzed by electrophoresis on a 1%–1.2% agarose gel (Invitrogen) containing ethidium bromide (Life Technologies), which was photographed under ultraviolet light.

### Fenton reaction

The reaction was performed in 20 µl containing pEGFP-C1 plasmid in PBS, Fe(NH_4_)_2_SO_4_ (Sigma-Aldrich), H_2_O_2_, and the other additives at the indicated concentrations. The mixture was incubated at 37°C for 1 hr and analyzed by 0.8% agarose gel electrophoresis.

### MALDI-TOF analysis

SDS-PAGE gels were stained with Coomassie Brilliant Blue solution (Amresco). For MALDI-TOF analysis, the stained target bands were carefully cut from the gel, destained, trypsinized, and dried. Before the sample/matrix solution dried, 0.5 µl (∼500 femtomoles of each calibrant) of calibration mixture from the Sequazyme peptide Mass standard kit (Applied Biosystems, Framingham, MA, USA) was added on top of each spot to provide internal calibrants. Once the spots on the target plate dried, the plate was introduced into the Voyager DE-STR MALDI-TOF mass spectrometer (Applied Biosystems) for analysis. The resulting mass spectra were calibrated by a two-point internal calibration with one of the trypsin autolysis peaks in the calibration mixture solution. The protein database was searched using the MASCOT program.

### Preparation of purified AFP

From MyBioSource (San Diego, CA, USA), we purchased recombinant human AFP purified from human placenta. Purified human AFP (2 µg/ml) was dissolved in storage buffer [0.1 mg/ml in 50 mM Tris-acetate, pH 7.5, 1 mM EDTA, 20% glycerol without bovine serum albumin (BSA) and sodium azide].

To prepare the bacterially purified GST-AFP, we used the Glutathione-S-Transferase (GST) Gene Fusion System (GE Healthcare Life Sciences). First, the complete coding sequence of the porcine *AFP* gene was cloned into the pGEX-4T-1 vector to generate N-terminal GST fusion constructs. The pGEX-4T-1-AFP construct was expressed in *Escherichia coli* BL21 cells, and the expression of GST-AFP was induced by treatment with 0.1 mM isopropyl-d-thiogalactopyranoside (IPTG; Sigma-Aldrich). The cells were harvested and resuspended in 30 ml of buffer A (100 mM NaCl, 50 mM Tris pH 8.0, 0.1% Triton X-100, 1 mM EDTA). The resuspended bacterial cells were sonicated with a sonicator (Bandelin Electronic GmbH & Co. KG, Berlin, Germany), and the lysates were centrifuged at 12,000 rpm for 15 min at 4°C. The supernatants were collected, and GST-AFP was purified using a Glutathione Sepharose 4B affinity matrix. Protein concentrations were determined by the Bradford assay (Bio-Rad) with BSA as the standard. The purified GST-AFP and GST proteins, together with molecular weight standards (Thermo Scientific Fermentas, Schwerte, Germany), were separated by 8% SDS-PAGE and stained with Coomassie Brilliant Blue.

### Hydrogen peroxide scavenging assay

The hydrogen peroxide scavenging potential of GST-AFP, GST, and estradiol was determined using the method described by Jayaprakasha et al. [23]. A solution of 40 mM hydrogen peroxide was prepared in PBS (pH 7.4). Different concentrations of GST (2 µg), GST-AFP (1, 2, and 4 µg), and estradiol (1, 5, and 10 nM) in PBS were added to 0.6 ml of the hydrogen peroxide solution. After 10 min of incubation, the absorbance was measured at 230 nm against a blank solution that contained hydrogen peroxide solution without protein or estradiol. The percentage of H_2_O_2_ scavenging was calculated as follows: % Scavenged [H_2_O_2_] = [(Abs control – Abs sample)/Abs control]×100.

### Statistical analysis

Results were expressed as the mean ± standard deviation (SD) of three independent experiments. Statistical analyses were performed using one-way ANOVA (analysis of variance) or a two-tailed Student's *t*-test in the Excel program (Microsoft, Redmond, WA, USA). Differences were considered significant when p<0.05.

## Results

### H-PE shows antioxidant effects in human keratinocytes

**Figure 1:**
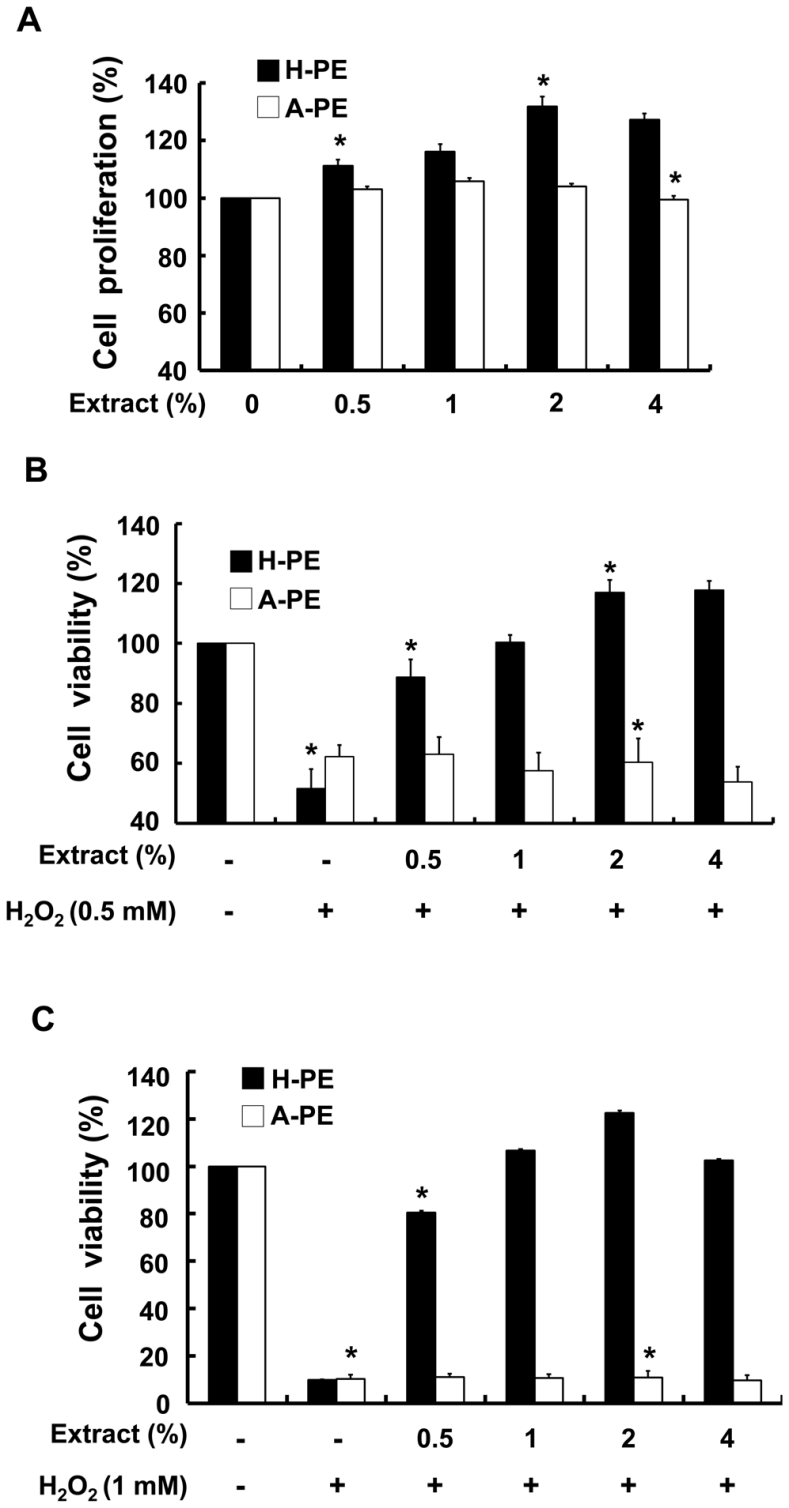
The proliferation and viability of human keratinocytes were analyzed after treatment with different concentrations of homogenized placental extract (H-PE) or acid-hydrolyzed placental extract (A-PE). Human keratinocytes (HaCaT) were incubated for 24 hr in the presence of different concentrations of (A) H-PE or A-PE; (B) H-PE or A-PE under 0.5 mM H_2_O_2_; and (C) H-PE or A-PE under 1 mM H_2_O_2_. Cell viability in the presence of H-PE or A-PE under oxidative stress was measured by the MTT assay. The data are presented as the percentage of control (non-treated cells) and the mean ± SD of at least three independent experiments. *p<0.05.

To investigate the antioxidant effects of the acid-hydrolyzed placental extracts (A-PE) or the homogenized placental extracts (H-PE) on human keratinocytes, human HaCaT cells were treated with different concentrations of A-PE or H-PE ranging from 0.5% to 4%. The proliferation of HaCaT cells increased with H-PE treatment, whereas no marked changes occurred in A-PE-treated cells (Figure 1A), indicating that porcine PE was not cytotoxic to human keratinocytes and that H-PE had a beneficial effect on the proliferation of keratinocytes.

To evaluate the effects of A-PE and H-PE on the viability of keratinocytes under oxidative stress, cells were exposed to different concentrations of H_2_O_2_. Under 0.5 mM and 1 mM H_2_O_2_ exposure, cell viability was reduced up to 50% and 10%, respectively (Figure 1B and 1C). However, when cells were exposed to 0.5 mM and 1 mM H_2_O_2_ in the presence of H-PE, cell viability improved nearly to the control viability level, whereas A-PE treatment had no obvious effect (Figure 1B and 1C), implying that H-PE, but not A-PE, contains antioxidant materials, which protected keratinocytes from oxidative-stress-induced cell death.

### H-PE treatment decreases H_2_O_2_-induced apoptotic and necrotic cell death

**Figure 2:**
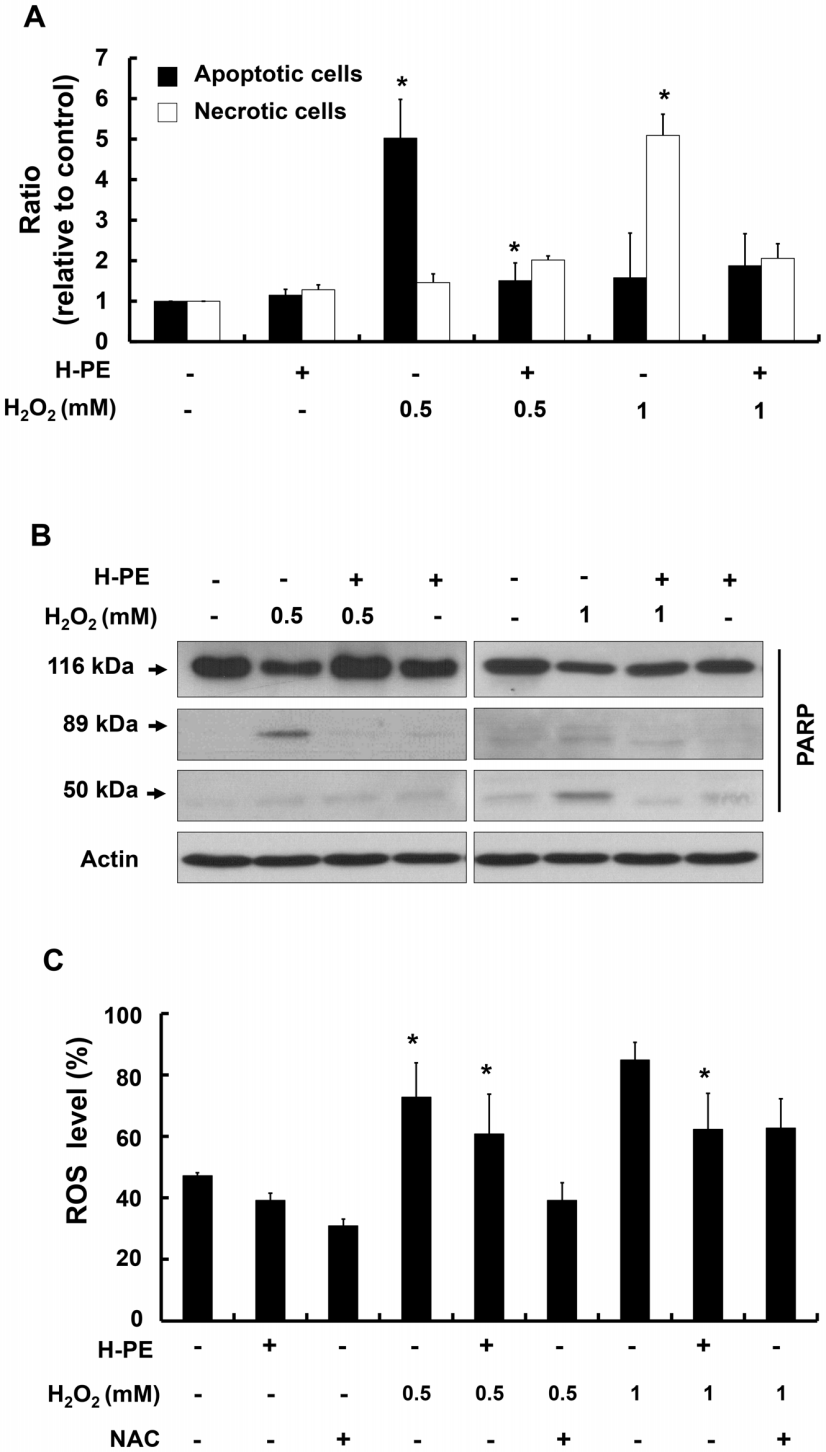
Apoptotic or necrotic cell death was studied under oxidative stress in the presence of H-PE. (A) The ratio of apoptotic and necrotic cell death in H-PE-treated HaCaT cells under oxidative stress was assessed by SYTOX-Hoechst 33342 staining. The relative ratios of apoptosis and necrosis are presented in the graphs. The graphs show the fold change relative to control (non-treated cells) and the mean ± SD (n = 3). *p<0.05. (B) Poly (ADP-ribose) polymerase (PARP) cleavage was observed by western blotting. The 89-kDa fragments of PARP represent apoptotic progression, whereas 50-kDa fragments appear in necrotic cells. (C) The ROS levels in HaCaT cells were measured by H_2_DCFH-DA staining and analyzed by FACS. Cells were treated with H-PE for 24 hr and exposed to different levels of oxidative stress (0.5 mM or 1 mM H_2_O_2_). H-PE treatment significantly (*p<0.05) reduced the ROS level under oxidative stress. N-acetylcysteine (NAC) was used as a positive control. The data are presented as a percentage of control (non-treated cells) and the mean ± SD from triplicate experiments.

To clarify the type of cell death induced by different H_2_O_2_ concentrations (0.5 and 1 mM), we examined cell death using the SYTOX-Hoechst staining method [24]. Apoptotic cells, with condensed or fragmented nuclei, were predominantly observed in 0.5 mM H_2_O_2_-treated cells, whereas necrotic cells were mainly observed in 1 mM H_2_O_2_-treated cells. When H-PE was added, apoptotic and necrotic cell death in H_2_O_2_-treated cells decreased (Figure 2A). These results were confirmed by observing PARP cleavage as a marker of apoptosis and necrosis: 89-kDa fragments represent apoptotic progression and 50-kDa fragments appear in necrotic cells [24]–[26]. We detected 89-kDa fragments in 0.5 mM H_2_O_2_-treated cells and 50-kDa fragments in 1 mM H_2_O_2_-treated cells. When cells were treated with H-PE, both the 89-kDa and 50-kDa fragments were reduced (Figure 2B), suggesting that H-PE treatment suppressed both H_2_O_2_-induced apoptotic and necrotic cell death in keratinocytes.

### The antioxidant component of H-PE is highly thermo-unstable

To validate the antioxidant property of H-PE, we compared intracellular ROS levels in the presence of H-PE or NAC, an antioxidant that is a reactive oxygen species scavenger [20]. ROS levels increased in cells treated with 0.5 mM or 1 mM H_2_O_2_. However, under H-PE treatment, the intracellular ROS level decreased, nearly to the level observed with NAC treatment (Figure 2C). These data indicated that H-PE had antioxidant properties and contributed to the reduction of H_2_O_2_-induced cell death by regulating ROS levels in keratinocytes.

**Figure 3:**
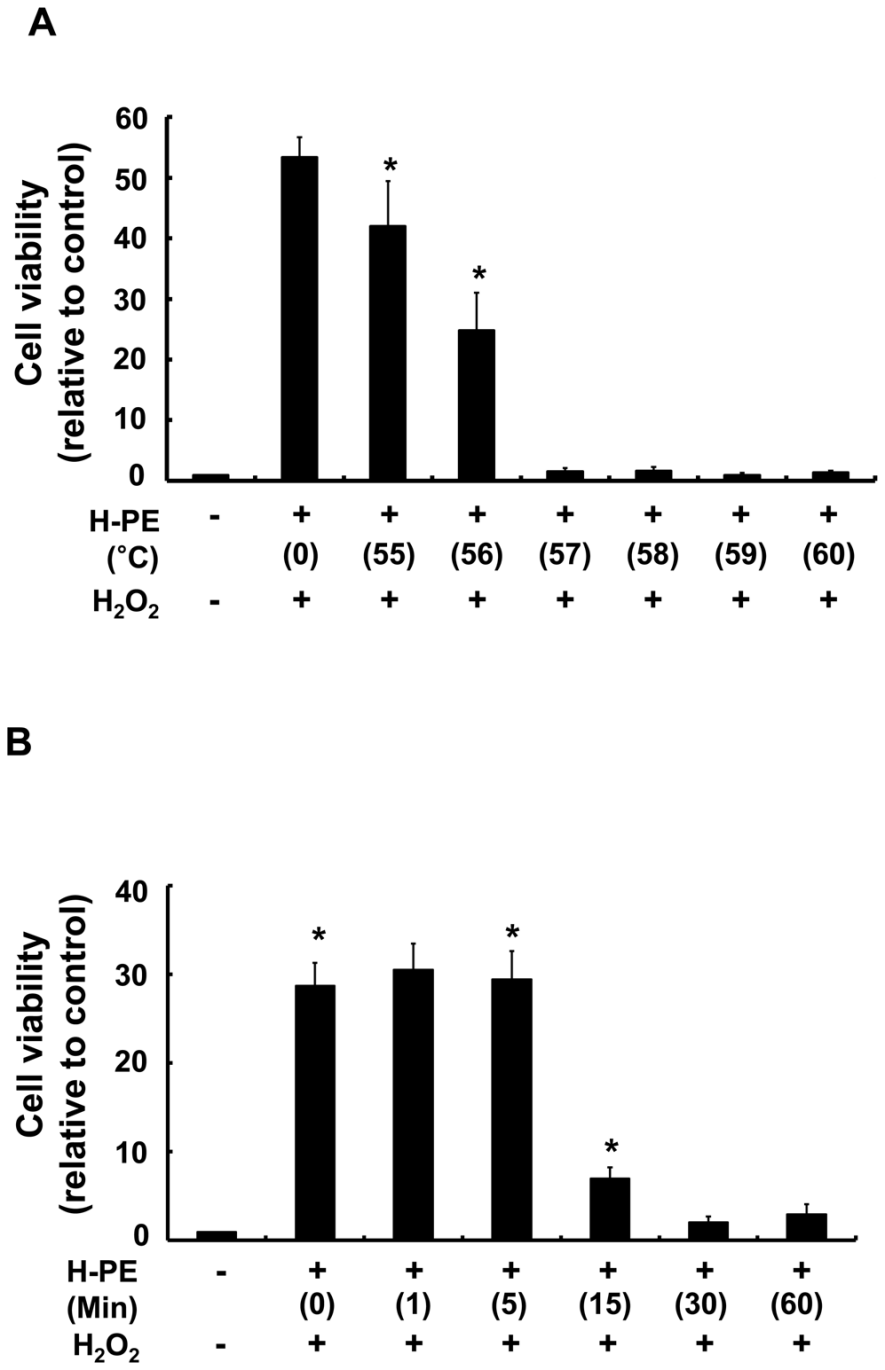
The thermo-stability of the antioxidant component of H-PE was assessed. (A) H-PEs were stored for 60 min at 55°C to 60°C, and their cell viability-rescuing effect was analyzed in H_2_O_2_-treated HaCaT cells. The graph shows the effect of heated H-PE on the cell viability of HaCaT cells. HaCaT cells were exposed to 1 mM H_2_O_2_ and cultured with H-PE heated at 55°C to 60°C (n = 3, *p<0.05). (B) HaCaT cells were treated with 1 mM H_2_O_2_ and cultured with H-PE heated at 60°C for 0 to 60 min. The cell viability was determined using the MTT assay. The data are presented as the percentage of control (non-treated cells) and the mean ± SD (n = 3, *p<0.05).

To analyze the thermo-stability of the antioxidant component in H-PE, H-PE was incubated for 60 min at 55°C to 60°C. The cell viability-rescuing effect was reduced starting from 55°C, and extracts incubated at over 57°C did not rescue cell viability in H_2_O_2_-treated HaCaT cells (Figure 3A). When the extracts were incubated at 60°C for longer than 30 min, the cell viability-rescuing effect was markedly reduced (Figure 3B), implying that the antioxidant component of H-PE is highly thermo-unstable. From these data, we predicted that the antioxidant components in H-PE were proteins, based on their sensitivity to heat.

### Fractionation and screening of the antioxidant protein in H-PE

**Figure 4:**
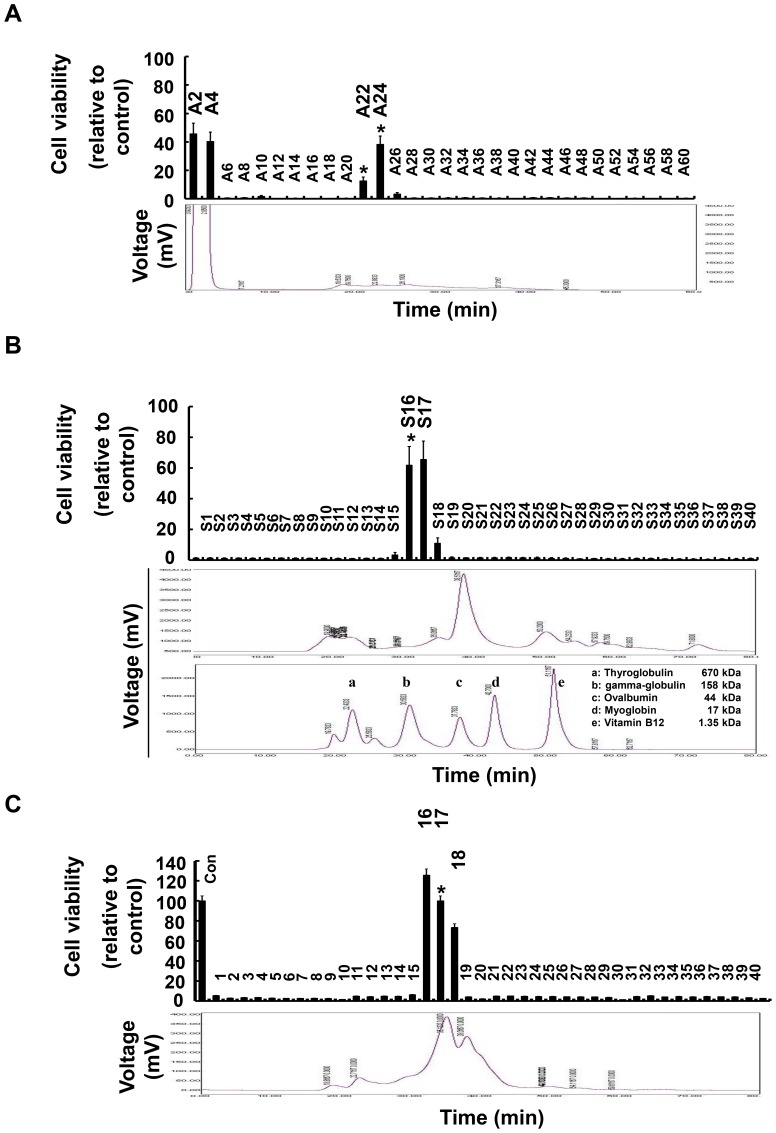
The cell viability of human keratinocytes was measured after treatment with H-PE fractions from anion-exchange and size-exclusion chromatography. H-PE was fractionated by (A) anion-exchange or (B, C) size-exclusion after anion-exchange chromatography. Using the MTT assay, the fractions were screened by estimating the viability of HaCaT cells treated with 1 mM H_2_O_2_ and each fraction for 24 hr. The cell viability indicated the antioxidant properties of the fractions. The values are presented as the percentage of control (non-treated cells) and the mean ± SD of triplicate experiments (*p<0.05). The chromatograms (second panels) show the elution curves of the proteins in H-PE. The second chromatogram (second panel in B) shows the elution curves of standard molecules: a. thyroglobulin, 670 kDa; b. r-globulin, 158 kDa; c. ovalbumin, 44 kDa; d. myoglobin, 17 kDa, e. vitamin B12, 1.35 kDa.

To identify the antioxidant components of H-PE, we fractionated H-PE using three types of chromatography (anion, cation, and size-exclusion chromatography). Keratinocytes were exposed to oxidative stress, and the cell viability in the presence of each fraction was investigated. First, H-PE was separated by cation- or anion-exchange chromatography; 60 cation and anion chromatographic fractions were collected. The antioxidant activity of each fraction was estimated from the viability of keratinocytes treated with H_2_O_2_. The cation-exchange chromatographic fractions C2–C4 increased cell viability (results not shown), and the anion-exchange chromatographic fractions A2–A4 and A22–A26 also increased cell viability (Figure 4A). Because early fractions might be flow-through, we determined that fractions A22–A26 contained antioxidant components.

Next, H-PE was separated by size-exclusion chromatography. Size-exclusion chromatographic fractions S16–S17 dramatically improved cell viability under H_2_O_2_ treatment (Figure 4B). According to the molecular weight of the standard proteins, fractions S16–S17 eluted in the range from gamma-globulin (158 kDa) to ovalbumin (44 kDa). Therefore, we presumed that the sizes of the antioxidant components were between 44–158 kDa. To determine whether fractions A22–A26 and S16–S17 contained the same antioxidant factors, the anion-exchange fractions (A22–A26) were separated by size-exclusion chromatography. The anion-exchange and size-exclusion double chromatography fractions had the same effect as fractions separated by size-exclusion chromatography alone (Figure 4C). The chromatography results indicated that anion-exchange fractions A22–A26 and size-exclusion chromatography fractions S16–S17 contained the same antioxidant components.

**Figure 5:**
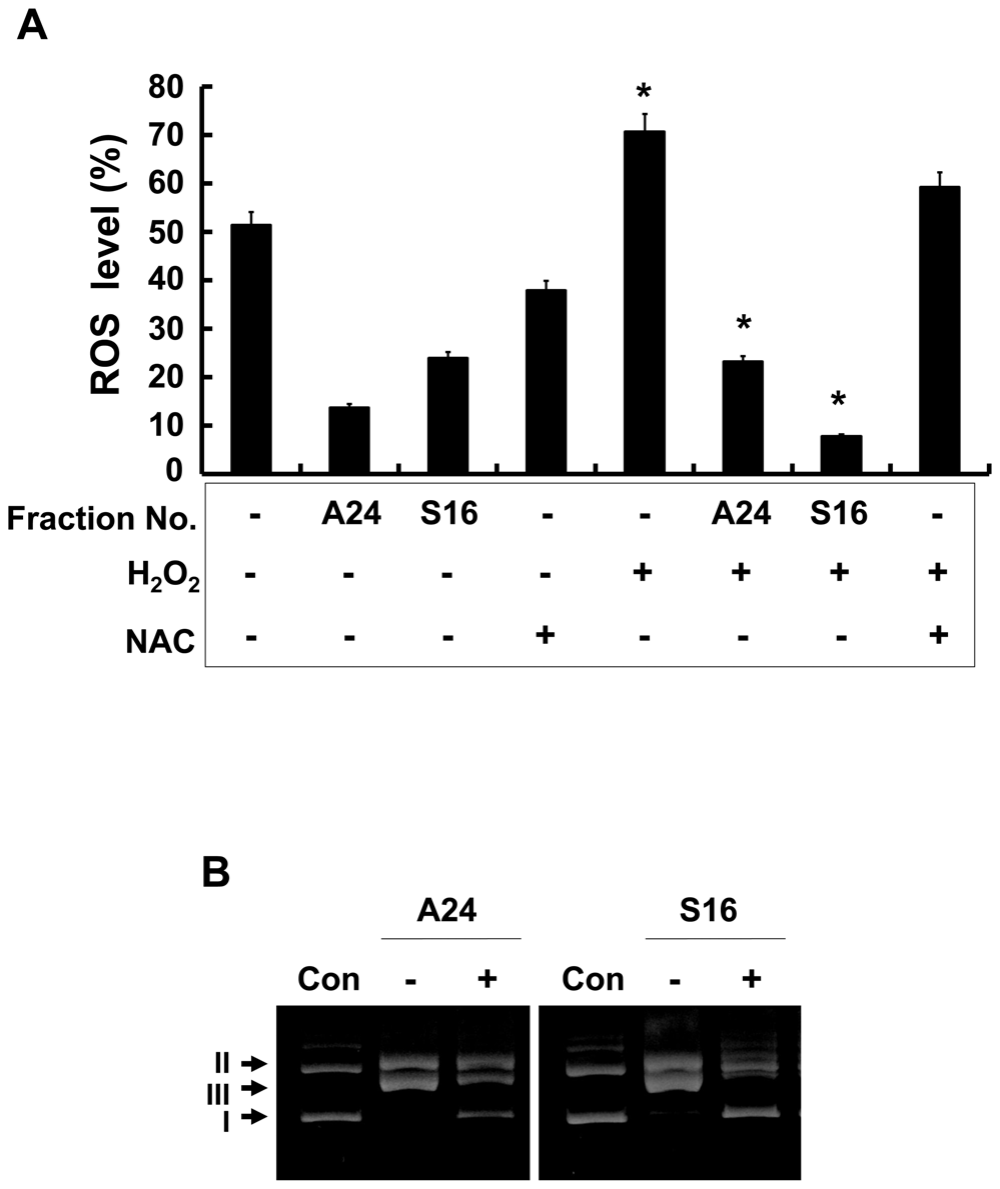
The antioxidant effects of the chromatographic fractions were analyzed. (A) The ROS levels in HaCaT cells treated with placenta-derived fractions were measured by the MTT assay. NAC was used as a positive control. The data are presented as the percentage of non-treated cells and the mean ± SD (n = 3, *p<0.05). (B) pEGFP-C1 plasmid was incubated at 37°C for 1 hr with the following: lane 1, no addition (plasmid only); lane 2, ferrous ammonium sulfate and H_2_O_2_; lane 3, ferrous ammonium sulfate, H_2_O_2_, and 4 µl of each indicated fraction. A, anion-exchange chromatographic fraction; S, size-exclusion chromatographic fraction.

To confirm the biochemical location of the antioxidant components, the effects of two selected fractions, A24 and S16, on H_2_O_2_-treated intracellular ROS levels in cells were estimated. ROS levels in keratinocytes treated with fractions A24 and S16 were statistically reduced (Figure 5A). The antioxidant properties of H-PE fractions A24 and S16 were further studied by the Fenton reaction. Generally, DNA stays in a super-coiled (I) form and an open circular (II) form. Fenton's reagent breaks the super-coiled form of DNA and generates the linear (III) form via a hydroxyl radical reaction [28]–[31]. As shown in lane 2 of Figure 5B, the plasmid was completely converted from the type I form to the type II and III forms by the Fenton reaction. In the presence of H-PE fractions A24 and S16, the super-coiled (I) form was protected from hydroxyl radical-induced DNA damage (Figure 5B). These results indicated that the antioxidant component was present in fractions A22–A26 and S16–S17.

### Alpha-fetoprotein is the main antioxidant marker in H-PE

**Figure 6:**
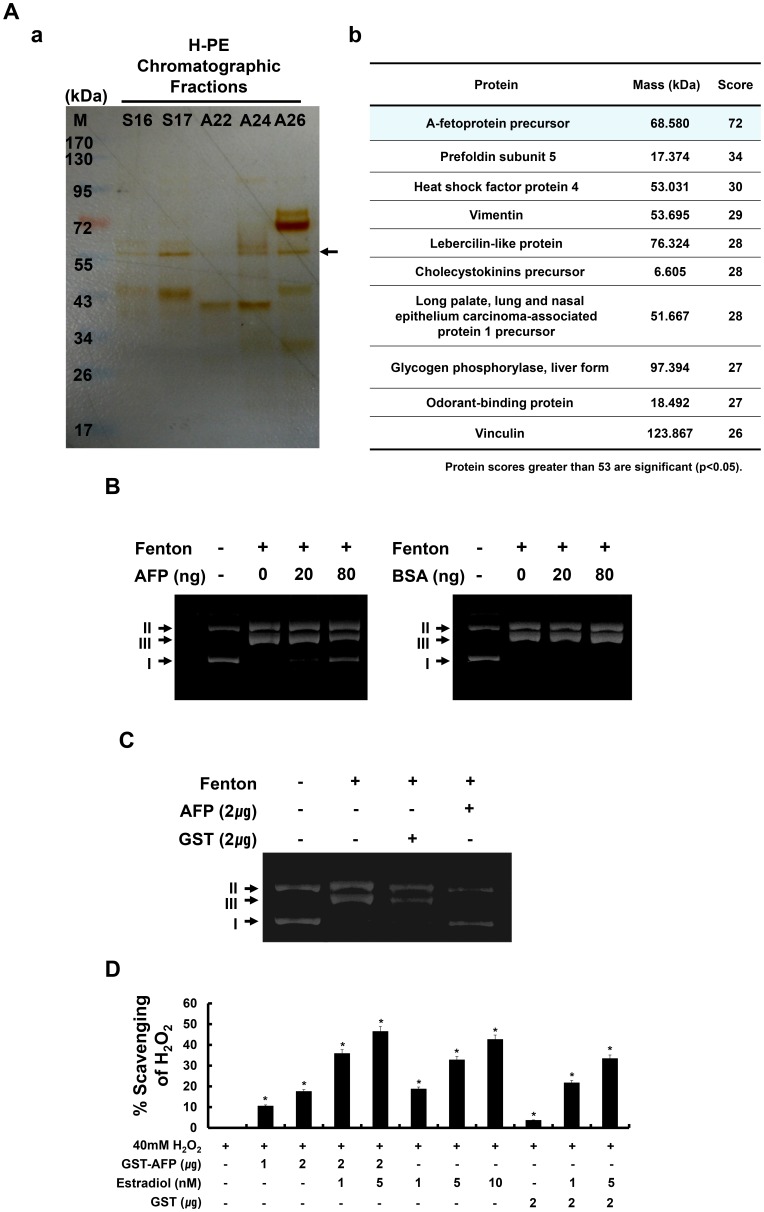
Alpha-fetoprotein (AFP) was identified as an antioxidant candidate in H-PE. (A) a, Proteins were visualized by silver staining. Arrows indicate AFP. The same fraction volume was loaded on the gel. Molecular masses are indicated on the left. M, protein marker. b, Profiles of candidate proteins by MALDI-TOF analysis. The MASCOT program was used to search the protein database. (B) The antioxidant effect of AFP was assessed from the I (super-coiled), II (open circular), and III (linear) plasmid patterns. Lane 1, no addition (plasmid only); lane 2, ferrous ammonium sulfate and H_2_O_2_; lanes 3 and 4, ferrous ammonium sulfate, H_2_O_2_, and human AFP (left panel). Bovine serum albumin (BSA) was used as a negative control (right panel). (C) The antioxidant effect of purified AFP and GST was investigated from the plasmid patterns. Lane 1, no addition (plasmid only); lane 2, ferrous ammonium sulfate and H_2_O_2_; lane 3, ferrous ammonium sulfate, H_2_O_2_, and purified GST; lane 4, ferrous ammonium sulfate, H_2_O_2_, and purified AFP. Purified GST was used as a negative control. (D) H_2_O_2_ scavenging results of purified GST, GST-AFP, and estradiol (%) (*p<0.05).

To identify the candidate antioxidant proteins, we compared the protein band patterns of the indicated fractions by SDS-PAGE (Figure 6A-a). Several bands were commonly observed, but one band had an intensity pattern similar to the mean value of cell viability. Candidate proteins were identified by MALDI-TOF and the MASCOT program. They included AFP precursor; pre-foldin subunit 5; heat-shock factor protein 4; vimentin; lebercilin-like protein; cholecystokinin precursor; and long palate, lung, and nasal epithelium carcinoma-associated protein 1 precursor. Among these, AFP precursor was chosen because of its protein scores, which were greater than 53 and statistically significant (Figure 6A–b). At the amino acid level, AFP precursor has 100% identity with AFP, and human AFP has 83% identity with porcine AFP.

The antioxidant property of AFP was confirmed by the plasmid patterns of forms I (supercoiled), II (open circular), and III (linear). As seen in lane 2 of the left panel of Figure 6B, the type I form was completely changed to the type II and III forms under the Fenton reaction without AFP. In the presence of 80 ng of AFP purified from human placenta, the supercoiled (I) form appeared in a dose-dependent manner (lanes 3 and 4, left panel, Figure 6B). In the presence of a control protein (80 ng BSA), the type II and III forms were present; no type I plasmid pattern was observed (lanes 2, 3, and 4, right panel, Figure 6B). Moreover, to confirm the antioxidant property of AFP, we performed the Fenton reaction using bacterially purified GST-AFP, with GST as a control (Figure 6C). In the presence of 2 µg of bacterially purified GST-AFP, the supercoiled (I) form appeared (lane 4, Figure 6C). However, in the presence of 2 µg of GST, the type I plasmid form was not observed (lane 3, Figure 6C). Furthermore, the antioxidant activity of bacterially purified GST-AFP was directly assessed using the hydrogen peroxide radical scavenging assay. Purified GST-AFP scavenged hydrogen peroxide in a dose-dependent manner (Figure 6D). The scavenging activity of purified GST-AFP (2 µg) was almost 20%. Interestingly, the scavenging activity of GST-AFP was synergistically enhanced in the presence of estradiol. However, in the presence of 2 µg of GST, almost no scavenging of hydrogen peroxide was observed (Figure 6D). Moreover, the scavenging activity of GST was not enhanced in the presence of estradiol. Previous studies showed that purified GST does not exhibit antioxidant properties [32]. Collectively, these data suggest that AFP in H-PE acts as an antioxidant protein.

**Figure 7:**
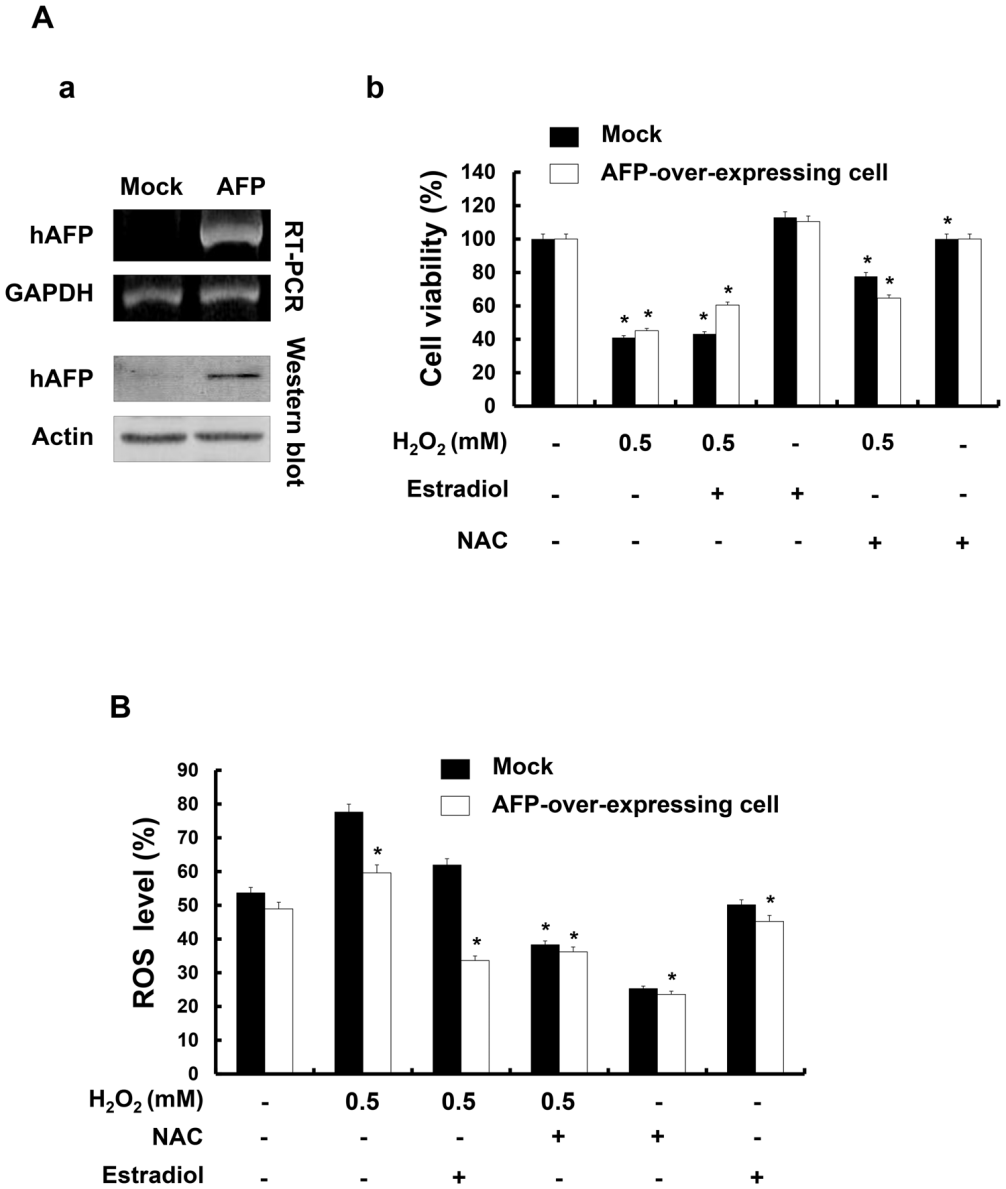
The effect of AFP overexpression was analyzed in HaCaT cells. (A) a, Overexpression of AFP was assessed in control HaCaT and AFP-overexpressing HaCaT cells by RT-PCR and western blot. Representative blots from three independent experiments are shown. b, Mock-transfected or AFP-overexpressing HaCaT cells were exposed to 0.5 mM H_2_O_2_, 5 nM estradiol, and 1 mM N-acetylcysteine (NAC). The cell viability was determined using the MTT assay. (B) ROS levels in mock-transfected or AFP-overexpressing HaCaT cells were measured by H_2_DCFH-DA staining and analyzed by FACS. Treatment with 5 nM estradiol significantly (*p<0.05) reduced the ROS level under oxidative stress. NAC was used as a positive control. The data are presented as the percentage of control (non-treated cells) and the mean ± SD from triplicate experiments.

Next, we assessed the effect of ectopic AFP overexpression on the cell viability of keratinocytes. First, we confirmed the exogenous expression of AFP in keratinocytes and verified that AFP levels increased in AFP-overexpressing cells (Figure 7A-a). Under 0.5 mM H_2_O_2_ exposure, cell viability was reduced up to 50% in mock-transfected (control) and AFP-overexpressing cells (Figure 7A-b). Although we detected slight increase in cell viability in AFP-overexpressing cells, treatment with NAC further lowered cell viability in AFP-overexpressing cells. The intracellular ROS level was also slightly decreased in AFP-overexpressing cells (Figure 7B). To investigate the synergic effect of estradiol and AFP on the antioxidant activity in keratinocytes under oxidative stress, we treated AFP-overexpressing cells with estradiol. When AFP-overexpressing cells were exposed to 0.5 mM H_2_O_2_ in the presence of estradiol, cell viability improved (Figure 7A-b). Estradiol treatment protected AFP-overexpressing keratinocytes from oxidative stress-induced cell death. Under estradiol treatment, the intracellular ROS level decreased significantly, nearly to the level observed with NAC treatment (Figure 7B). These data indicated that estradiol had antioxidant properties and protected against H_2_O_2_-induced cell death by regulating ROS levels in AFP-overexpressing keratinocytes. Taken together, these data suggest that AFP is a potential antioxidant agent and a novel marker protein for the antioxidant effect of H-PE.

## Discussion

Many studies have investigated the mechanisms of various materials with the potential for use in clinical or cosmetic fields. Some of these studies focused on extracts derived from natural substances [16], [33], [34]. Porcine PE has been reported to accelerate wound healing in the skin of rats, indicating that PEs might be useful for skin protection [6]. Although human PE contains anti-oxidative, collagen-derived peptides [5], [9], their effects at the cellular level have not been extensively investigated. Several studies showed the effects of PEs on various cellular activities, such as anti-inflammatory, melanogenesis, wound healing, and antioxidant responses [17], [18], [34]–[36]. However, the active components of PE were not identified.

In our study, we prepared porcine extracts to confirm that H-PE has antioxidant effects in H_2_O_2_-treated human keratinocytes. Our results indicated that porcine placenta was a good source material for a strong antioxidant (Figure 2). To identify the main component with strong antioxidant activity in PE, we separated H-PE by chromatography and tested the antioxidant effect of several fractions (Figures 3 and 4). Protein bands in the selected chromatography fractions were compared by silver staining. AFP precursor was identified as a candidate protein by MALDI-TOF and the MASCOT program (Figure 6). During fetal development, AFP, a serum glycoprotein, is produced at high levels in the liver and the yolk-sac endoderm and at lower levels in the developing gastrointestinal tract [37], [38]. AFP synthesis decreases dramatically after birth, and only trace amounts are detected in adults [38]. The abundance of AFP during fetal development has led to speculation that this serum protein is essential for mammalian embryo development and/or sexual differentiation [35], [36], [39]. AFP also binds and transports numerous ligands, including bilirubin, fatty acids, retinoids, steroids, heavy metals, dyes, flavonoids, phytoestrogens, dioxin, and various drugs [40], [41]. Abnormal levels of embryonic AFP are indicative of spina bifida or Down's syndrome in the fetus [42], [43]. Several groups have reported that AFP participates in various biological activities, such as drug conjugation, ligand binding, carrier functions, and growth regulation [14], [15]. However, the function of AFP in adults is not clear. The current results suggest a novel role for AFP as an antioxidant and identify AFP as a marker protein for the antioxidant effect of porcine placenta.

Several studies have suggested that the binding of estrogen to AFP is related to antioxidant activity [44]–[46]. Estrogens, especially 17-beta-estradiol, which possess a phenolic hydroxyl group, have antioxidant activity. Estradiol may affect several anti-atherogenic genes, such as nitric oxide synthase [47], thereby increasing the levels of nitric oxide, an antioxidant [21], [22], [48]–[51]. When AFP-overexpressing keratinocytes were incubated with estradiol and 0.5 mM H_2_O_2_, we observed a synergistic antioxidant effect (Figure 7B). To determine the function of AFP *in vivo*, further studies are necessary. Taken together, our data suggest that AFP is a potent antioxidant agent and a novel marker protein for the antioxidant effect of H-PE. Thus, AFP or the AFP-containing H-PE fraction may have applications in skin protection.
